# Biliary mucinous cystic neoplasm: a case report and review of the literature

**Published:** 2016-12

**Authors:** Mohammad Taghi Safari, Shabnam Shahrokh, Mohammad Bagher Miri, Forough Foroughi, Amir Sadeghi

**Affiliations:** 1 *Gastroenterology and Liver Diseases Research Center, Research Institute for Gastroenterology and Liver Diseases, Shahid Beheshti University of Medical Sciences, Tehran, Iran*; 2 *Basic and Molecular Epidemiology of Gastrointestinal Disorders Research Center, Research Institute for Gastroenterologyand Liver Diseases, Shahid Beheshti University of Medical Sciences, Tehran, Iran*

**Keywords:** Biliary mucinous cystic neoplasm, Hepatobiliary cystadenoma

## Abstract

Hepatobiliary cystadenomas (HBC) is a rare neoplasm which comprising less than one percent of liver cystic neoplasms. Although it’s known as a benign tumor, but they have a potential for neoplastic transformation. Making a proper diagnosis and ruling out of other differential diagnosis is important because of different treatment. In the present study, we described a case of HBC manifested as idiopathic dominant biliary stricture in common hepatic duct (CHD), on the basis of spiral CT scan and MRI, and elevated CA19-9. With a probable diagnosis of malignant biliary stricture, she underwent ERCP and cholangioscopy that were non-diagnostic and final diagnosis was made surgically. HBCs usually found incicentally as a cystic lesion and biliary stricture without visible cyst in imaging like that seen in cholangiocarcinoma is very unlikely. In truth, this patient is an unusual manifestation of one rare disease.

## Introduction

Hepatobiliary cystadenomas (HBCs) and cystadenocarcinomas are rare lesions which comprise one percent of liver cystic neoplasms and 5% of symptomatic hepatic cysts (1,2). In approximately 85% of cases the lesions usually develop in liver and these lesions are very rare in extrahepatic bile ducts and gallbladder (3-7). Based on tumor cell types and tumor stroma, biliary cystic neoplasms are heterogeneous. In regards of cell types they can be mucinous or non- mucinous and according to tumor stroma they classified as presence or absence of ovarian like stroma (3,8- 11). HBCs, is a benign cystic neoplasm with unknown etiology, although the congenital abnormalities of the biliary tract is reported to be associated with the entity [(3). It occurs mostly in women and found incidentally. Although it is known as a benign lesion, several studies reported the malignancy association of this lesions as well (3-6-8). Differential diagnosis of cystadenoma from cystadenocarcinoma is difficult preoperatively, therefore, surgical resection is highly recommended (9). On the other hand, several different modalities have been introduced to manage HBC including simple aspiration, drainage, enucleation, marsupialization, and complex excision of cystadenoma. The lack of definite presentations and symptoms beside normal laboratory findings in majority of cases make it difficult to distinguish HBC from other cystic lesions of the liver. To prevent the recurrence and malignant progression of HBCs, total surgical excision of the tumors is highly recommended (12). So the diagnosis of HBC is crucial in order to avoid further complications. In the present study we described an extreme rare case of HBC characterized by the presence of a benign mucinous tumor in common hepatic duct, which is known as biliary mucinous cystic neoplasms (BMCNs) of the liver.

## Case report

A 32-year-old female referred to our center with progressive generalized pruritus and jaundice, which had been started 4 months before the admission time. She also noted the loss of appetite which followed by 5 kg weight loss over the preceding four months. She did not have any fever, nausea, diarrhea, anorexia, abdominal pain, rash, respiratory symptoms, arthralgia or clay-colored Stools. However, the color of the urine became over concentrated. The patient did not indicate the history of traveling, consumption of alcohol, herbal medicine, antibiotics or any other drugs. She had been well until 4 months ago without remarkable habit, family or past medical history. Physical examination revealed an icteric patient with excoriated skin. There were no other significant physical findings including thyroid, heart, lung and abdomen. Laboratory data revealed elevated alkaline phosphatase, inflammatory parameter and CA19-9 with normal serum aminotransferases, total bilirubin, prothrombin time and serum albumin level. Alcohol, acetaminophen, and other toxicology screens were negative. Serum creatinine and blood urea nitrogen were normal, and urinalysis did not reveal blood or leukocytes. (table 1).

**Table 1:** Laboratory values

Hemoglobin (g/dL)	12.7
Leukocytes (× 103 U/L)	10.3
Platelets (× 103 U/L)	315
Total bilirubin (mg/dL)	5.5
Direct bilirubin (mg/dL)	3.2
AST (IU/L)	25
ALT (IU/L)	29
Alkaline phosphatase (IU/L)	601
ESR	63
CRP	2+
Total protein (mg/dL)	7.2
Albumin (mg/dL)	3.9
Prothrombin time (s)	12
CA19-9	405

**Figure 1:**
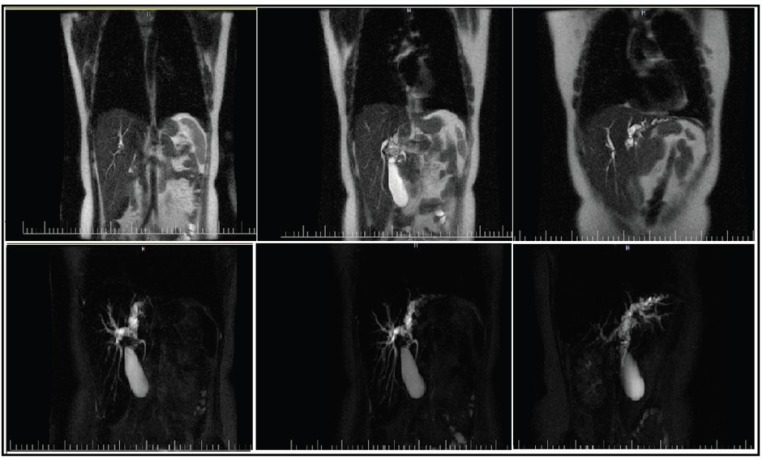
MRCP (magnetic resonance cholangiopancreatography) showed dilatation of intrahepatic ducts along with suspicious stenosis of CHD

Ultrasound of the abdomen revealed dilation of intrahepatic bile ducts of the left and right lobe without evidence of hepatosplenomegaly, cirrhosis, or ascites. Triphasic spiral CT scan of the abdomen confirmed mild dilatation of intrahepatic ducts without any pathologic lesion and MRCP images showed dilatation of intrahepatic ducts along with suspicious stenosis at common hepatic duct (CHD) (figure 1).

The patient then underwent ERCP and cholangioscopy. In cholangioscopy, partial obstruction probably due to external compression was detected in common hepatic duct without any evidence of ulceration or infiltrative lesion (Seefigure 2). Cholangiographic guided biopsy and brush cytology were non-diagnostic.

Thepatientreferredforsurgery.Intraoperative cholangioscopy revealed small bile duct cystic lesion with compression of common hepatic duct. Finding of surgery was a 3*5cm cystic mass in proximal of CBD with extension to CHD and right hepatic duct and a 5*5 cystic mass in segment III of liver which had luminal communication with the bile duct and periportal lymphadenopathy. The bile duct neoplasm and liver cyst were resected and following lymph node dissection, a Roux-en-Y hepaticojejunostomy was conducted. Intraoperative frozen section was then diagnosed as low grade mucinous cystic lesion of common bile duct. It was consisted of an irregular multi-cystic tan brown elastic tissue measuring 2.3x0.6x0.3 (figure 3). Microscopy showed a multi-cystic structure lined by a single layer of the mucinous type epithelial cells with basally- located bland-looking nuclei. The underlying stroma revealed ovarian-like pattern. No mitosis was observed. A liver cyst was also sent for permanent diagnosis which showed the same microscopic features as mentioned above. Enlarged periportal lymph nodes were reactive (figure 3). The patient showed no recurrence after 12 months of follow-up.

**Figure 2:**
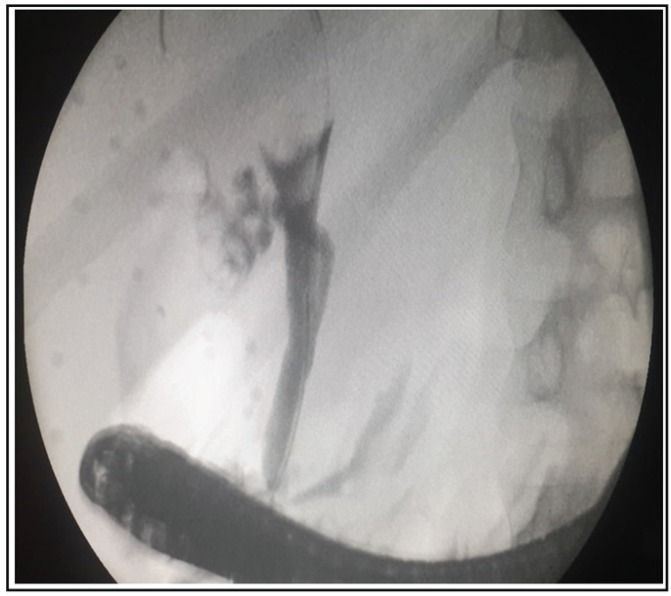
ERCP (endoscopic retrograde cholangiopancreatography) showed partial obstruction in CHD probably due to external compression

**Figure 3:**
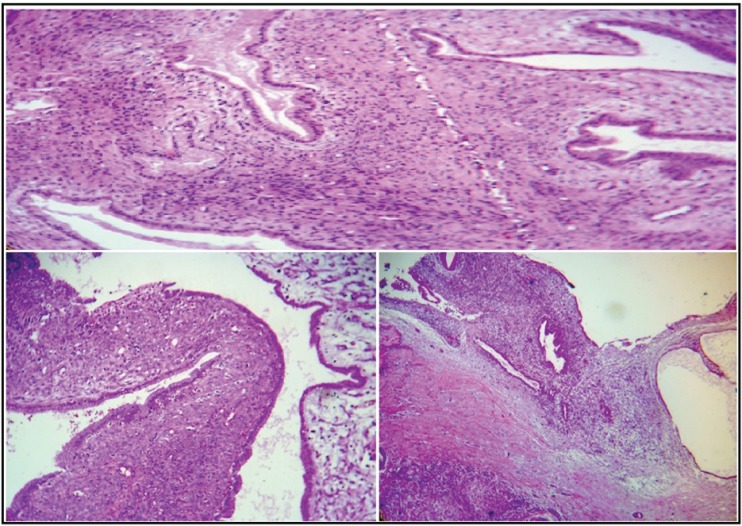
Histologic evaluation showed multi-cystic structure lined by a single layer of the mucinous type epithelial cells with basally- located bland-looking nuclei

## Discussion

Biliary cystadenomas account up to 5% of intrahepatic cystic lesions of biliary origin. Among hepatobiliary cysts, mucinous cystic neoplasms are very rare with an incidence rate of 1 in 20 000–100 000 for non-invasive types (13). In the present study we reported an extreme rare case of a 32-year-old female of HBC characterized by the presence of a benign mucinous tumor in common bile duct. In line of our study, Paradis, et al. described a case of a 57-year-old female presenting with jaundice related to hepatobiliary cystadenoma. Histopathological evaluation confirmed the diagnosis of HBC. The patient underwent left hepatectomy with cholecystectomy and intrabiliary tumoral bud resection (14). In another study, Sang, et al. evaluated 33 cases with hepatobiliary cystadenomas and cystadenocarcinomas (15). They revealed that clinical symptoms are not reliable for differential diagnosis of these cases from each other. They also found that imaging modalities and CA19-9 are valuable only for detection of cystadenoma and cystadenocarcinoma and not for their differential diagnosis. It has been demonstrated that the treatment in both non-invasive and invasive cases of biliary mucinous cystic neoplasms is surgical approach. However, the resectability of tumors is highly dependent on the tumour site and functional liver reserve (6,16,17). Complete resection of cystic adenoma is the gold standard and regarding as the best approach without the risk of recurrence in patients (6), Since several approaches used for treatment of biliary cystic adenoma including internal Roux- en-Y drainage, sclerosis, aspiration, or partial resection have been reported to be associated with high rates of recurrence (5,13,18). In a review study by Simo etal. on biliary mucinous cystic neoplasms (BMCNs), indicated that since the patients symptoms and laboratory findings are not sufficient to make an accurate diagnosis of the BMCNs, complete resection of suspected non-invasive or invasive lesions is the best treatment option in order to avoid further complications(1). In this regard, Emre, etal.also reported that even considering the recent development of imaging approaches for detection of biliary cystadenoma from cystadenocarcinoma, definite preoperative diagnosis may be complicated. Hence, complete surgical excision of the suspected lesion is the best treatment choice in related patients (19). Enucleation is an alternative approach when the resection of the tumor is difficult or there is a risk of morbidity in patient (2,20). In present case report, the bile duct neoplasm and liver cyst were resected. Intraoperative colangioscopy was performed and following lymph node dissection, hepaticojejunostomy was conducted. Intraoperative biopsy and frozen section are vital for the diagnosis of the lesions (21). Wang, et al. study on 20 cases with biliary cystadenoma and 10 cases with biliary cystadenocarcinoma showed that, older age, male gender, and shorter symptom duration are correlated with a higher risk of biliary cystadenocarcinoma (22). In addition, Yoh Zen et al. (23) also reported that hepatic mucinous cystic neoplasms and biliary intraductal papillary neoplasms have both distinct clinicopathological characteristics based on age and gender of patients, macroscopic appearance, immunophenotypes, and grades of malignancy. The malignancy potential of non- invasive lesions in case of transforming to invasive type has been also reported in several studies (24,25). Approximately 75% of patients with benign cystadenomas are older than 30 years. The patients may have no presentations and discovered incidentally. Whereas, the most common symptom is abdominal pain. The majority of mucinous cystic neoplasms develop in the liver, except for rare tumours in the gallbladder or common bile duct (3,10). In conclusion, hepatobiliary cystadenomas have malignancy potential and may accompany with some related presentations. Although clinical symptoms are not reliable for the diagnoses of cystadenomas alone and even the presence of imaging modalities are not able to differentiate between benign and malignant cysts. Hence, complete surgical resection of suspicious noninvasive or invasive cystadenomas is the best treatment option in such cases to prevent further complications and enhance the patients’ survival.
